# Role of Positron Emission Tomography in Imaging of Non-neurologic Disorders of the Head, Neck, and Teeth in Veterinary Medicine

**DOI:** 10.3389/fvets.2019.00180

**Published:** 2019-06-11

**Authors:** Mathieu Spriet, Jennifer L. Willcox, William T. N. Culp

**Affiliations:** Department of Surgical and Radiological Sciences, School of Veterinary Medicine, University of California, Davis, Davis, CA, United States

**Keywords:** tumor, neoplasia, staging, inflammation, pain, dog, cat, computed tomography

## Abstract

Positron Emission Tomography (PET) is an imaging technique that provides functional information, in addition to structural information obtained with computed tomography (CT). The most common application is cancer staging, using ^18^F-Fluorodeoxyglucose (^18^F-FDG), a radioactive analog of glucose. Although limited data are available in the veterinary literature, human studies have demonstrated benefit with the addition of PET both for assessment of the primary tumor and for detection of metastatic disease. ^18^F-FDG PET appears to be more accurate at detecting the margin of oral neoplasia, in particular for tumors arising from highly vascularized tissue, such as the lingual and laryngeal areas. ^18^F-FDG PET has a high sensitivity for the detection of lymph node metastasis, however the specificity is variable between studies. Tracers beyond ^18^F-FDG can also be used for oncology imaging. ^18^F-Fluoride (^18^F-NaF) is an excellent osseous tracer, useful in assessing bone involvement of primary tumors or osseous metastasis. Other specific tracers can be used to assess cell proliferation or hypoxia for tumor characterization. ^18^F-FDG is also an excellent tracer for detection of inflammation. Human studies have demonstrated its value for the assessment of periodontitis and dental implant infection. ^18^F-NaF has been used to assess disorders of the temporomandibular joint in the human literature, demonstrating good correlation with arthralgia and therapeutic outcome. Both ^18^F-NaF and ^18^F-FDG had good concordance with localization of cervical pain in people. PET will likely have a growing role in veterinary medicine not only for oncologic imaging but also for assessment of inflammation and pain.

## Introduction

Positron Emission Tomography (PET) is a nuclear medicine imaging technique, which provides cross-sectional data based on the 3-dimensional localization of positrons emitted by radiotracers. When compared with scintigraphy, in addition to its cross-sectional nature, PET has the advantages of higher detection efficiency and spatial resolution (1). PET data reflects the distribution of the radiotracer in the area being imaged, but does not provide structural information relating to the anatomy of the patient. Rather, PET provides functional information based on the interaction of the radiotracers at the molecular level in relation to physiological events. In order to facilitate anatomic localization of areas of increased radiotracer uptake, PET is usually combined with computed tomography (CT), which provides structural information of the patient. Quantification of the radiotracer uptake is commonly reported in PET studies using the maximal standardized uptake value (SUVmax), which is calculated based on the injected dose corrected for decay at the time of imaging and weight of the patient.

A broad choice of radiotracers is available for PET. 18F-Fluoride is the most commonly used positron emitter. The 2-h half-life is convenient for clinical use and fluoride can be easily integrated into various organic molecules in place of a hydroxyl group. The most commonly used radiotracer is 18F-Fluorodeoxyglucose (^18^F-FDG), which is a radioactive analog of glucose. Similar to glucose, FDG is actively transported into cells by a group of structurally related glucose transport proteins. For this reason, ^18^F-FDG is an excellent marker of metabolic activity and is particularly useful for the detection of neoplasia, as tumor cells often preferentially utilize glucose for glycolysis and subsequently display increased numbers of glucose transporters (2). Cancer staging is by far the most common application of PET imaging using ^18^F-FDG. However, ^18^F-FDG uptake is not only found in neoplastic tissue but also in areas of inflammation (3).

Another commonly used tracer is 18F-Fluoride (^18^F-NaF). ^18^F-NaF is an excellent marker of bone remodeling as it gets integrated into the exposed hydroxyapatite matrix at sites of bone turnover. ^18^F-NaF has a higher sensitivity than the larger and more complex 99mTc-Bisphosphonates used for scintigraphic imaging. ^18^F-NaF has been commonly used in oncology for the detection of primary osseous tumors or metastasis but can also be used for non-oncologic imaging (4).

PET availability remains limited in the veterinary field, with only a few academic institutions offering clinical PET imaging, but there is a growing interest in the technique based on recent publications. Similar to the human field, oncologic applications largely dominate the clinical use of PET, but other indications are being considered.

## Oncologic PET Imaging of Head and Neck

**Figure 1:**
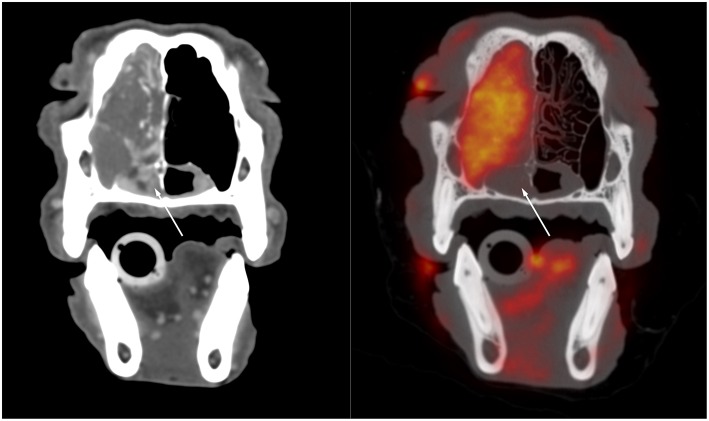
Transverse post-contrast CT **(left)** and fused 18F-FDG PET/CT **(right)** images through the caudal aspect of the nasal cavity of a 10-year-old male castrated standard poodle presented for staging of nasal adenocarcinoma. There is a large soft tissue mass filling the left side of the nasal cavity and resulting in destruction of the nasal turbinates and invasion of the maxillary recess. The soft tissue opacity extends ventrally into the rostral aspect of the nasopharynx. The opacity in the nasopharynx is strongly contrast enhancing on the CT image but does not show FDG uptake whereas the rest of the mass demonstrates strong FDG uptake. This suggests that the tissue in the nasopharynx differs from the bulk of the tumor and might represent edematous nasopharyngeal mucosa rather than neoplastic tissue.

The veterinary literature describes the use of PET for staging of several canine and feline tumors. One study examined oral squamous cell carcinoma in 12 feline patients (5, 6). The oral tumors were more conspicuous on PET than on contrast CT (5). PET also identified hypermetabolic tissue considered to be potentially neoplastic outside of the suspected neoplastic area recognized with CT (5). This was more commonly identified in patients with smaller ill-defined lesions on CT, in particular if their tumors arose from the lingual, pharyngeal, and laryngeal regions rather than the mandibular and maxillary areas (6). Interestingly, the measured tumor volume was smaller on PET than on contrast CT in the majority of patients. (6) This is similar to observations in a human study that demonstrated a smaller volume of pharyngolaryngeal squamous cell carcinoma measured with FDG PET when compared with CT and MRI, with a difference in volume ranging from 28 to 37%. Surgical correlation demonstrated that all imaging modalities overestimated the size of the tumor, but PET was the most accurate modality (7). The increased accuracy in definitions of the margin is of particular importance for treatment planning, both for surgery or radiation therapy. Better margin definitions are more likely to yield to successful surgical outcomes and improve targeted delivery of radiations. An example of the comparison between CT alone and fused FDG PET with CT in a patient with nasal carcinoma imaged at our institution using a high-resolution PET scanner (piPET, BrainBiosciences, Inc.) is provided in Figure 1. The difference observed between contrast CT and ^18^F-FDG PET can be explained by the type of information provided. Contrast CT highlights areas with increased perfusion, whereas ^18^F-FDG PET identifies areas with high metabolic activity. ^18^F-FDG PET can be advantageous in areas that are normally highly vascularized where contrast CT is less likely to demonstrate a difference between a tumor with increased blood perfusion and the highly vascularized background (6).

In addition to ^18^F-FDG PET, ^18^F-NaF PET has been suggested for assessment of bone involvement in cancer (4). A comparative study between ^18^F-FDG and ^18^F-NaF PET demonstrated a superior correlation with histopathology for ^18^F-NaF. (8) Further characterization of tumor biological activity can be achieved using specific tracers. This is of particular interest to optimize treatment planning. For example, 18F-deoxyfluorothymidine (^18^F-FLT) and ^61^Cu-diacetylmethylthiosemicarbazone (^61^Cu-ATSM), are markers of tumor proliferation and hypoxia, respectively, which are useful indicators of tumor sensitivity to radiation (9). Twenty dogs with sinonasal tumors were imaged with ^18^F-FDG, ^18^F-FLT, and ^61^Cu-ATSM. Heterogeneity with regard to proliferation and hypoxia of the tumors was demonstrated. Carcinomas demonstrated strong correlations between the standardized uptake values of the three tracers, whereas sarcomas were less likely to correlate (10). ^18^F-fluoro-misonidazole (^18^F-miso) is another marker of hypoxia used in human studies (11) with potential applications in veterinary medicine (12).

PET/CT has been used extensively in the detection of local and distant metastasis. In the feline oral squamous cell carcinoma study, only limited cytologic confirmation of metastasis was available, however out of three cytologically confirmed lymph node metastasis, two were identified based on PET findings, but were not apparent on contrast CT (5). To our knowledge, larger studies looking at the accuracy of PET for detection of lymph node metastasis are lacking in the veterinary literature. Human literature often concludes a higher accuracy of PET compared with CT alone regarding metastasis detection but a wide range of results have been reported (13–18). This suggests that tumor type, tumor locations, and imaging techniques likely affect outcome. There is an overall trend toward higher sensitivity of PET for metastasis detection when compared with CT and MRI, but some studies identify a lack of specificity (13, 17). PET findings should be interpreted in conjunction with clinical presentation and other imaging findings to optimize the accuracy and reduce false positives. Species and tumor specific studies are needed in the veterinary literature to elucidate the value of PET and whether it could be considered a less invasive alternative to lymph node sampling.

## Non-Oncologic PET Imaging of Head and Neck

There are limited reports evaluating the use of PET in non-oncologic applications in the veterinary literature. The most common use of PET for the detection of inflammation relates to neurologic disease (19), but ^18^F-FDG PET was also useful in a case of Blastomyces dermatitis, as well as cases with a fever of unknown origin (20, 21). Orthopedic uses of PET have recently gained interest in veterinary medicine. The use of ^18^F-FDG has been proposed for lameness evaluation in a dog (22). Recently, PET has been introduced to equine lameness imaging with the use of ^18^F-NaF PET for assessment of active bone remodeling (23–25).

**Figure 2:**
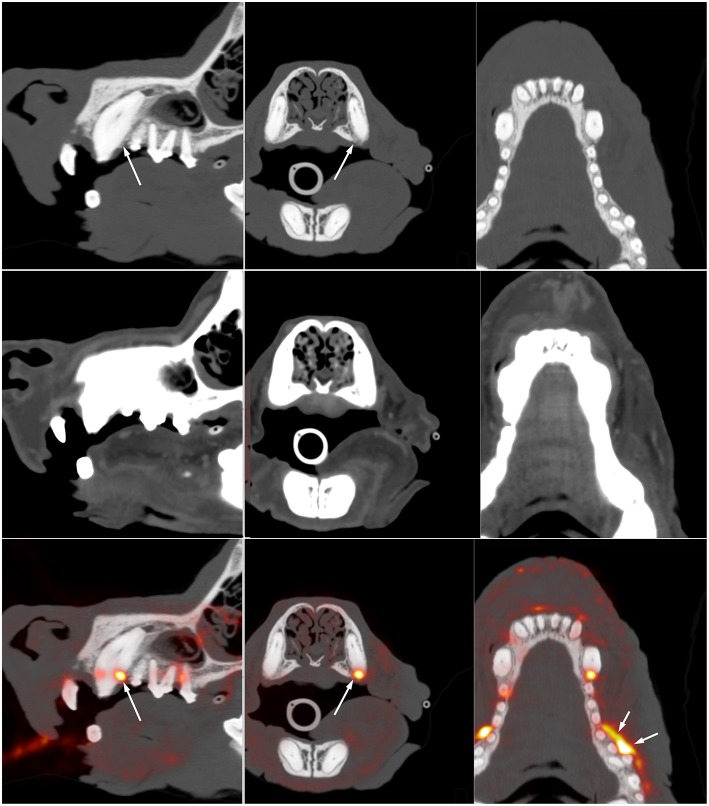
Multiplanar reformat CT **(top)** and fused 18F-FDG PET **(bottom)** images through the maxilla of a 12-year-old Australian Cattle Dog presenting for staging of a previously resected oral melanoma. There is vertical and horizontal bone loss involving the left canine and left maxillary premolars 1, 2, and 3. Marked focal ^18^F-FDG uptake (long arrow) is present at the distal aspect of the left maxillary canine. Additional resorptive areas are present adjacent to the three most mesial premolars, the lack of ^18^F-FDG uptake in this area suggest that there is no active inflammation at this level. The marked ^18^F-FDG uptake at the buccal aspect of premolars 3 and 4 is at the site of previous resection of an oral melanoma and likely indicate regrowth or residual tumor tissue. This case illustrates the sensitivity of ^18^F-FDG PET to distinguish between active and inactive periodontal disease and to identify abnormal tissue at the site of previous tumor resection. It also demonstrates the lack of specificity and the need to interpret the images based on associated CT findings and clinical history. Note that the high spatial resolution (~2 mm) on the compact scanner (piPET, Brain Biosciences, Inc) is key in identifying these small lesions.

In the human literature, there are various interesting applications for non-oncologic PET imaging of the head and neck. A few reports have assessed the value of ^18^F-FDG PET for identification of periodontal disease or apical periodontitis (26–28). Although ^18^F-FDG PET had not been performed for the primary purpose of periodontal assessment, these studies demonstrated that useful information regarding the oral cavity can be obtained when patients are imaged with PET for other indications. Similar observations exist in canine patient. Figure 2 is an example of a dog imaged with high-resolution PET for tumor assessment and demonstrates active periodontal disease. A recent study in people investigated the use of PET for assessment of dental implants and concluded that functional imaging using ^18^F-FDG could become a new tool for the assessment of peri-implant diseases (29).

^18^F-NaF PET has been suggested for assessment of temporomandibular joint disorders (30, 31). Previous studies in human medicine have demonstrated a superior ability to detect osteoarthritis of the temporomandibular joint when compared with 99mTc-MDP bone scintigraphy (30). Furthermore, SUVmax appeared to correlate with arthralgia and therapeutic outcome (30). ^18^F-NaF PET imaging has also been used for assessment of neck pain. Although the high background vertebral uptake can be a limitation, PET was still considered beneficial in a study for 84.5% of the 58 patients with neck pain (32). ^18^F-FDG PET also appeared useful as guidance for therapy in patients with cervical facet syndrome. In a study involving 140 facet joints, 100% concordance was found between location of focal ^18^F-FDG uptake and painful area in the neck (33).

In conclusion, PET is best known for its use in oncologic staging, however the array of radiotracers available provide a diverse set of applications. The functional properties of the technique apply to not only the metabolic activity of tumors but also the assessment of bone remodeling, inflammation, and pain. With changes in technology, including the decreased cost and the availability of high resolution PET scanners, it is likely that the use of PET in veterinary medicine will experience continued growth in the future.

## Ethics Statement

The images presented in this manuscript were obtained as part of clinical trial projects approved by the animal care and use committee and the clinical trial review board of the University of California Davis.

## Author Contributions

MS redaction of the manuscript. JW and WC contribution to redaction of the manuscript. MS, JW, and WC approval of final manuscript.

### Conflict of Interest Statement

The authors declare that the research was conducted in the absence of any commercial or financial relationships that could be construed as a potential conflict of interest.
